# Patient-reported outcome measures for non-specific neck pain validated in the Italian-language: a systematic review

**DOI:** 10.1186/s40945-016-0024-2

**Published:** 2016-07-22

**Authors:** Leonardo Pellicciari, Francesca Bonetti, Damiano Di Foggia, Mauro Monesi, Stefano Vercelli

**Affiliations:** 1 Program in Advanced Sciences and Technologies in Rehabilitation and Sports Medicine, Tor Vergata University, Rome, Italy; 2 Department of Clinical Sciences and Translational Medicine, Tor Vergata University, Rome, Italy; 3 Department of Rehabilitation, Rome American Hospital, Rome, Italy; 4 Department of Neuroscience, Rehabilitation, Ophthalmology, Genetics, Maternal and Child Health, University of Genoa - Campus of Savona, Savona, Italy; 5 Laboratory of Ergonomics and Musculoskeletal Disorders Assessment, Division of Physical Medicine and Rehabilitation, Salvatore Maugeri Foundation, Scientific Institute of Veruno, IRCCS, Veruno, NO Italy

**Keywords:** Outcome assessment, Quality of life, Spine, Pain, Disability evaluation

## Abstract

**Background:**

Patient-reported outcome measures can improve the management of patients with non-specific neck pain. The choice of measure greatly depends on its content and psychometric properties. Most questionnaires were developed for English-speaking people, and need to undergo cross-cultural validation for use in different language contexts. To help Italian clinicians select the most appropriate tool, we systematically reviewed the validated Italian-language outcome measures for non-specific neck pain, and analyzed their psychometric properties and clinical utility.

**Methods:**

The search was performed in MEDLINE, EMBASE, CINAHL, Scopus, Web of Science, and Cochrane Library. All articles published in English or Italian regarding the development, translation, or validation of patient-reported outcome measures available in the Italian language were included. Two reviewers independently selected the studies, extracted data, and assessed methodological quality using the COSMIN checklist.

**Results:**

Out of 4891articles screened, 66 were eligible. Overall, they were of poor or fair methodological quality. Four instruments measuring function and disability (Neck Disability Index, Neck Pain and Disability Scale, Neck Bournemouth Questionnaire, and Core Outcome Measures Index), and one measuring activity-related fear of movement (NeckPix©) were identified. Each scale showed some psychometric weaknesses or problems with functioning, and none emerged as a gold standard.

**Conclusions:**

Several patient-reported outcome measures are now available for assessing Italian people with non-specific neck pain. While the Neck Disability Index is the one most widely used, the Neck Bournemouth Questionnaire appears the most promising tool from a psychometric point of view.

## Background

Non-specific neck pain (NSNP) has a multifactorial etiology and it is frequently associated with psychosocial disorders such as anxiety or depression [1]. NSNP affects about two-thirds of people at some stage in their life, especially in middle age [2]. Reliable and valid patient-reported outcome measures (PROMs) can provide useful information for a more appropriate prognosis and management. The selection of a PROM greatly depends on its content (the construct being measured), and the soundness of its psychometric properties. These include reliability, validity, responsiveness, interpretability of scores, quality of translation, and acceptable patient/investigator burden [3].

Several instruments are currently available to assess patients affected by NSNP. A recent review [4] concluded that there was no need for the development of new questionnaires, but rather for more information on the measurement properties of the existing instruments. In most cases, these tools were developed and validated in English-speaking populations. To adapt them to a different language context, a cross-cultural translation process using well-accepted methodological standards is required. In 2011, a systematic review [5] of non-English versions of NSNP questionnaires pointed out that the only instrument validated in the Italian language was the Neck Pain and Disability Scale (NPDS). However, in the last 5 years other instruments have been translated or newly developed in Italian, and further studies carried out on the NPDS.

The aim of this study was to systematically review the psychometric properties and clinical utility of the validated Italian-language PROMs available to assess patients affected by NSNP, with the intention of helping clinicians to select the most appropriate scale for their needs.

## Methods

### Search strategy and study selection

A structured search of MEDLINE, CINAHL, EMBASE, Scopus, Web of Science, and Cochrane Library databases was performed from their inception to November 2015. Search strategies for all databases are reported in Appendix. All peer-reviewed articles published in English or Italian that made reference to the development, validation, or clinical use of PROMs to assess patients with NSNP were considered. Other descriptive articles (reviews, clinical trials, letters, commentaries, etc.) that did not provide psychometric data, as well as studies including subjects with specific neck pain (i.e. myelopathy, radiculopathy, whiplash-associated disorders), were excluded.

Three reviewers (FB, DDF, and MM) independently screened titles and abstracts to exclude duplicates and obviously irrelevant studies. The electronic search was complemented by a hand search of the reference list of retrieved articles for additional relevant studies. Disagreements between reviewers were resolved by consensus. Afterwards, two reviewers (LP and SV) independently extracted data on the PROMs available in Italian. For an in-depth understanding of their psychometric properties, data were also collected for any other language version of selected instruments.

### Quality assessment

Methodological quality assessment of the studies included was performed with the COnsensus-based Standards for the selection of health Measurement INstruments (COSMIN) checklist [6]. In the COSMIN checklist ten boxes can be used to assess whether a study meets the standards for good methodological quality. Nine of these boxes contain standards for the included measurement properties and were rated in this review (the Box for criterion validity was excluded as no gold standard exists for neck pain PROMs). Each box consists of different items, that are rated individually on a 4-point rating scale (i.e. “poor”, “fair”, “good” or “excellent”, see http://www.cosmin.nl). Subsequently, an overall score for the assessment of a given measurement property is obtained by taking the lowest score for any of the items in the box (‘worst score counts’ method). In addition, the generalizability box was used in a data extraction form: information about the characteristics of the study sample in which the measurement properties were assessed are included in the tables related to each scale. Assessment of methodological quality was carried out by two reviewers (LP & SV) independently. In the case of disagreement, a consensus was obtained through discussion and a third reviewer (FB) gave the score. When the terminology used in the included studies was uncertain, the COSMIN consensus-based definitions of measurement properties were used to decide which properties were assessed and the corresponding boxes to tick.

### Data extraction and analysis

Two authors independently extracted data regarding language, sample size, and studied population. After the assessment of methodological quality with the COSMIN checklist, relevant data on the psychometric properties of reliability, validity, and responsiveness based on classical test theory (CTT) were extracted and interpreted using the following methods [3].

Reliability includes internal consistency and test-retest reliability [7]. The internal consistency is the level of interrelatedness between each item or between items and the total score. A positive rating for internal consistency was given when factor analysis was applied, and Cronbach’s alpha was between 0.70 and 0.95 [7]. A low Cronbach’s alpha indicates a lack of correlation between the items, which makes summarizing them unjustified, while a very high value indicates redundancy of one or more items [7]. Test-retest reliability concerns the degree to which several measurements made at different times provide similar scores, considering the fact that the clinical condition remains stable. As a general guideline, Intraclass Coefficient Correlation (ICC) values above 0.75 are indicative of good reliability, and those below 0.75 poor to moderate reliability. However, for most clinical measurements reliability should exceed 0.90 in order to ensure reasonable validity [8].

The most common approach used for validation of an instrument is factor analysis [8]. A factor represents a subset of items that are related to each other - but not to items in other factors - reflecting a single theoretical component of the construct (unidimensionality). Unidimensionality of a PROM is a necessary prerequisite to calculate a composite total score. When available, the factor analysis for each PROM was discussed. The construct validity of a scale could be evaluated also in terms of how its score correlates to other measures of the same (convergent validity) and different (divergent validity) constructs [7, 9]. Pearson or Spearman correlations were categorized as strong if ≥0.70, moderate if 0.50–0.69 and weak if 0.26–0.49 [10].

Responsiveness is the ability of a measure to detect within-person changes over time. Distribution and anchor-based methods are the two general approaches used to interpret score changes and to calculate the Minimal Clinically Important Difference (MCID), also known as the Minimal Important Change [11]. The MCID should be based primarily on anchor-based procedures (Receiver Operating Characteristic [ROC] curves are the preferred approach) [12]; it should be higher than Minimum Detectable Change (MDC) values (the boundary of variability typically found in stable patients) [12, 13]; and it should not be based on one study or method only [14]. The ROC curve gives the optimal cut-off value (usually the point that jointly maximizes sensitivity and specificity, associated with the least amount of misclassification) and the Area Under the Curve (AUC). The greater the AUC, the greater a measure’s ability to distinguish patients who have improved from those who have not improved. As a rule, AUC values between 0.7 and 0.8 are considered as acceptable, and an AUC value higher than 0.8 has a good to excellent discriminative capacity [15]. Among the distribution-based methods, the most useful index is the MDC, i.e. the smallest change in score that is beyond random error. This value represents the statistical significance of individual changes and is expressed in the same metric as the scale. Other indices - such as Effect Size (ES), Standardized Response Mean (SRM), or Guyatt’s Responsiveness Index (GRI) - are frequently interpreted with Cohen’s thresholds: >0.80 large; >0.50 moderate; >0.20 small [8].

When available, the results of more powerful statistical approaches such as Rasch analysis (RA) were reviewed. Instruments that fit the Rasch model fulfill the requirements for the main mathematic manipulations of the scores, which is a key aspect when measuring clinical changes. RA is being increasingly used in the development and evaluation of PROMs in order to test whether the properties of a questionnaire comply with a wide range of psychometric requirements, such as assessment of response format, item content, appropriate targeting, reliability, and so on [16–18]. RA is used also to provide further confirmation of a scale’s unidimensionality. To confirm unidimensionality, a cut-off of 50 % of the variance explained by the Rasch factor (latent trait), and an eigenvalue of the first residual factor <3 are usually required conditions [19].

## Results

### Study selection

**Fig. 1:**
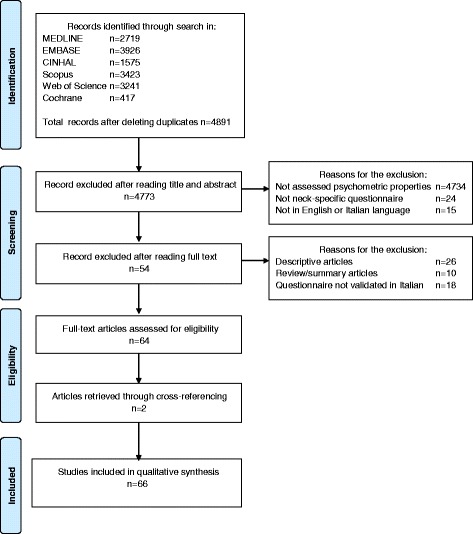
Flow-chart of study selection

A total of 4891 articles were initially identified in the literature search. Of these, 118 full-text articles were retrieved and 64 met the inclusion criteria. Two additional articles were found by hand searching. Therefore, a total of 66 articles were included in this systematic review for data collection. A flow chart of the selection process is reported in Fig. 1.

A total of 5 scales or questionnaires were identified: the Neck Disability Index (NDI), the Neck Pain and Disability Scale (NPDS), the Neck Bournemouth Questionnaire (NBQ), the Core Outcome Measures Index (COMI), and NeckPix®.

### Quality assessment

A detailed methodological assessment of the studies included in the review is reported in Table 1. Overall, most of the psychometric properties were classified as of low (poor to fair) methodological quality. The most common methodological shortcomings found were inadequate sample size included in the analysis, missing information (e.g. percentage of missing items not reported, no description of how missing items were handled), and methodological limitations of specific psychometric properties (i.e. not formulating a priori hypotheses regarding correlations or mean differences, or the direction of correlations or mean differences concerning the hypotheses testing; not complying with all the required translation steps for cross-cultural validity; not formulating a priori hypotheses about the changes in scores and the expected direction of correlations or mean differences of the change scores of PROM regarding responsiveness). Excellent rating was given to only a few boxes, and it was mostly related to the characteristics of internal consistency or validity. A comparison of how instruments validated in Italian language performed with respect to those validated in other languages was not possible owing to the very limited data available on Italian instruments. Cross-cultural validation processes were mainly conducted by a single workgroup. Generally the methodological quality of the translation process was low [20–22], except for the study on NBQ which was good [23]. However, the Italian studies added relevant insights with some good to excellent quality assessment rating, such as for the responsiveness box in the study by Monticone et al. [24].

**Table 1:** Assessment of methodological quality of the included studies using the COnsensus-based standards for the selection of health measurement instruments checklist. Where the psychometric properties were not included in the studies, the boxes are left blanks

Authors, year	Internal consistency	Reliability	Measurement error	Content validity	Structural validity	Hypotheses testing	Translation process	Responsiveness
Hains et al, 1998 [53]	Excellent				Good	Good		
Stratford et al, 1999 [54]	Fair	Poor	Poor		Poor	Poor		Poor
Wheeler et al, 1999 [65]	Poor			Poor	Fair	Fair		
Chok & Gomez, 2000 [26]		Poor						Poor
Ackelman & Lindgren, 2002 [27]		Poor		Poor		Poor		
Bolton & Humphreys, 2002 [76]	Poor	Poor	Poor			Poor		
Goolkasian et al, 2002 [73]		Poor				Poor		
Wlodyka-Demaille et al, 2002 [28]		Poor			Fair	Fair	Poor	
Bicer et al, 2004 [69]	Poor					Poor	Poor	
Bolton, 2004 [80]								Poor
White et al, 2004 [81]		Fair		Poor		Fair		
Wlodyka-Demaille et al, 2004 [75]								Poor
Lee et al, 2006 [29]	Poor	Poor				Poor	Poor	Poor
Vos et al, 2006 [30]		Fair	Fair					Poor
Gay et al, 2007 [59]	Poor					Poor		Poor
Kose et al, 2007 [31]	Poor	Fair				Poor	Poor	Poor
Mousavi et al, 2007 (NDI) [32]	Fair	Fair		Poor			Poor	
Mousavi et al, 2007 (NPDS) [32]	Fair	Fair		Poor	Fair		Poor	
Pool et al, 2007 [63]			Fair					Fair
Cleland et al, 2008 [60]		Fair	Fair					Fair
Kovacs et al, 2008 [82]	Poor	Poor				Poor	Poor	Poor
Monticone et al, 2008 [20]	Fair	Fair			Fair	Poor	Poor	
Scherer et al, 2008 [67]	Excellent				Good	Good	Poor	
Trouli et al, 2008 [33]	Good	Poor	Poor		Good		Fair	Fair
Chan et al, 2009 [74]				Poor		Poor		
Martel et al, 2009 [77]		Poor				Fair	Poor	Fair
Telci et al, 2009 [36]		Fair				Poor	Poor	
van der Velde et al, 2009 [57]	Fair				Fair	Poor		
Young et al, 2009 [61]		Poor	Poor					Good
Andrade Ortega et al, 2010 [34]	Fair	Poor	Poor		Fair	Fair		Fair
Jorritsma et al, 2010 (NDI) [70]		Poor		Poor				
Jorritsma et al, 2010 (NPDS) [70]		Poor	Poor	Poor			Fair	
Salo et al, 2010 [37]	Excellent	Poor			Good	Poor	Poor	
Wu et al, 2010 (NDI) [38]	Poor	Poor				Poor	Fair	
Wu et al, 2010 (NPDS) [38]	Poor	Poor			Poor	Poor	Fair	
Blozik et al, 2011 [72]			Poor					Poor
Chen et al, 2011 [68]	Good	Fair			Good	Poor	Fair	
Odole et al, 2011 [35]		Fair		Poor				
Ono et al, 2011 [71]	Fair	Fair			Fair			Poor
Pickering et al, 2011 (NDI) [55]					Fair			
Pickering et al, 2011 (NPDS) [55]					Poor			
Shakil et al, 2011 [39]	Poor	Fair					Fair	
Uthaikhup et al, 2011 [40]	Good	Poor	Good	Poor	Good	Poor	Fair	
Kesiktas et al, 2012 [41]	Fair	Fair			Fair	Fair	Fair	
Jorritsma et al, 2012a [62]	Poor			Poor		Good		
Jorritsma et al, 2012b [64]								Poor
Luksanapruksa et al, 2012 [42]	Poor	Fair			Poor	Poor	Poor	
Monticone et al, 2012 [21]	Excellent	Fair	Good	Poor	Excellent	Good	Fair	
Nakamaru et al, 2012 [43]	Poor	Fair			Fair	Poor	Fair	Poor
Soklic et al, 2012 [78]	Poor	Poor	Poor			Poor	Poor	
Ailliet at al, 2013 [44]				Excellent	Fair		Good	
Guzy et al, 2013 [45]	Fair	Fair	Fair		Fair	Poor	Poor	Poor
Johansen et al, 2013 [46]					Good	Poor		
Shaheen et al, 2013 [47]	Poor	Fair			Fair		Fair	Poor
Walton & MacDermid, 2013 [58]		Fair	Fair		Fair	Poor		Poor
Cramer et al, 2014 [48]	Fair	Poor			Fair	Poor		Poor
Geri et al, 2014 [23]	Good			Fair	Good	Good	Good	Good
Johansen et al, 2014 [89]	Poor	Fair	Fair					Poor
Miekisiak et al, 2014 [83]		Good	Good		Excellent	Poor		
Monticone et al, 2014 [22]		Good	Good			Good	Poor	Poor
Monticone et al, 2014 [84]	Excellent	Good		Poor	Excellent	Good		
Swanenburg et al, 2014 [52]	Poor	Fair	Fair		Poor	Poor	Fair	
Ailliet et al, 2015 [90]		Fair	Fair					Poor
Bakhtadze et al, 2015 [51]	Excellent	Good	Good		Excellent	Poor	Fair	
Cruz et al, 2015 [49]	Good	Good			Good	Poor	Fair	
Geri et al, 2015 [79]	Fair				Fair			
Hung et al, 2015 [56]					Fair			
Joseph et al, 2015 [50]	Poor	Fair		Fair		Poor	Fair	
Monticone et al, 2015 [24]								Excellent
Pereira et al, 2015 [91]			Fair					Fair

### Data extraction and analysis

Among the 66 studies included in this review, seven were conducted in Italy. Data regarding language, sample size, and studied population were classified by instrument and are reported in Tables 2, 3, 4, 5 and 6. The most studied psychometric parameters were reliability and validity, while less than half of the studies addressed measurement error and responsiveness. The overall low (poor to fair) quality of the studies and the heterogeneity of statistical approaches used prevented the use of a structured analysis relating results on specific parameters of each instrument to the study’s quality. Hence, only a descriptive synthesis of data was possible for each of the five instruments.

**Table 2:** Psychometric properties of the neck disability index

Authors, year (language)	Population	Sample size	Dimensionality	Internal consistency	Reliability	Validity	Responsiveness	
							Distribution-based methods	Anchor-based methods
Hains et al, 1998 (English) [53]	NP	237	1 factor	α = .92	–	r_p_ = .65 VAS-P	–	–
Stratford et al, 1999 (English) [54]	NP	50	1 factor	α = .87	ICC = .94 (1-sided lower 95 % CI .87)	r = .70 prognostic ratings of change	MDC = 4.7 points SEM = 2 points	MCID = 5 points AUC = .90 (Spec .80; Sens .78)
Chok & Gomez, 2000 (Malaysian) [26]	NP	22	–	–	k = .90	–	–	–
Ackelman & Lindgren, 2002 (Swedish) [27]	Chronic NP >3 months	39	–	–	r_s_ = .94–.99	r_s_ = .95 DRI r_s_ = -.88 SF-36 PF subscale r_s_ = .86 VAS-A r_s_ = .60 VAS-P	–	–
Wlodyka-Demaille et al, 2002 (French) [28]	Neck disorders >15 days	101	2 factors: Function and disability; Neck pain	–	ICC = .93	r_s_ = .88 NPQ r_s_ = .79 NPDS r_s_ = .54 HADS-D r_s_ = .50 VAS-D r_s_ = .48 VAS-P r_s_ = .43 HADS-A r_s_ = -.41 ROM (Flex-Ext)	–	–
Wlodyka-Demaille et al, 2004 (French) [75]	Neck disorders >15 days	71	–	–	–	–	ES = .55 SRM = .55	–
Lee et al, 2006 (Korean) [29]	NP	180	–	α = .92	ICC_2,1_ = .90 (95 % CI .81–.94)	–	ES = 1.04 (84 % CI .94–1.13) SRM = 1.17 (84 % CI 1.06–1.28)	AUC = .79 (95 % CI .72–.86)
Vos et al, 2006 (Dutch) [30]	First time or recurrent NP	79	–	–	ICC = .90 (95 % CI .82–.95) LoA = -7.40 - + 7.92	–	MDC = 1.66 points SEM = 0.60 points Guyatt = 1.82	
Gay et al, 2007 (English) [59]	NP >3 months	23	–	α = .72–.77	–	r_s_ = .77–.80 NBQ r_s_ = .44–.45 VAS-P	ES = 1.12 SRM = 1.21	–
Kose et al, 2007 (Turkish) [31]	NP >6 weeks	102	–	α = .90	ICC = .86	r = .71 VAS-D r = .65 VAS-P r = .58 Pain with palpation r = .50 Pain with motion r = -.41 ROM (Flex-Ext) r = -.41 ROM (Rot)	SRM = .85–.86	–
Mousavi et al, 2007 (Iranian) [32]	NP	185	–	α = .88	ICC = .97	r_p_ = .86 NPDS r_p_ = .71 VAS-P r_p_ = -.70–-.36 SF-36 subscales	–	–
Pool et al, 2007 (Dutch) [63]	NP and/or stiffness >2 weeks	183	–	–	–	–	MDC = 10.5 points	MCID = 3.5 points (Spec .70; Sens .90)
Cleland et al, 2008 (English) [60]	NP	138	–	–	ICC_2,1_ = .50 (95 % CI .51–.87) r_p_ = .56	–	MDC = 19.6 points SEM = 8.4 points	MCID = 9.5 points (Spec .72; Sens .83) AUC = .83 (95 % CI .75–.90)
Trouli et al, 2008 (Greek) [33]	NP	65	1 factor	α = .85	ICC = .93 (95 % CI.84–.97)	–	MDC = 1.78 points SEM = .64 points	–
Chan et al, 2009 (English) [74]	Chronic NP	20	–	–	–	r_p_ = .86 NPDS r_p_ = .62 PET	–	–
Telci et al, 2009 (Turkish) [36]	Chronic NP	88	–	–	ICC = .98 (CI 95 % = .97–.99)	r_p_ = .62 VAS-P r_p_ = .73 NPDS	–	–
van der Velde et al, 2009 (English) [57]	Mechanical NP	521	RA: unidimensionality is achieved by removing items #3 lifting, and #5 headache	–	–	NDI-8: r_s_ = .42 Pain	–	–
Young et al, 2009 (English) [61]	Mechanical NP	91	–	–	ICC_2,1_ = .64 (95 % CI.19–.84)	–	MDC = 10.2 points SEM = 4.3 points	MCID = 7.5 points AUC = .79 (95 % CI .68-.89)
Andrade Ortega et al, 2010 (Spanish) [34]	NP	175	1 factor	α = .89	ICC = .98 (95 % CI.98–.99)	r_p_ = .89 NPQ r_p_ = .65 VAS-P	–	–
Jorritsma et al, 2010 (Dutch) [70]	NP >3 months	32	–	–	ICC = .84 (95 % CI.69–.92 LoA = ±6.5	–	–	–
Salo et al, 2010 (Finnish) [37]	NP	101	1 factor	α = .85	ICC = .94 (95 % CI.90–.96)	r_p_ = .53 VAS-P r_p_ = .43 DEPS	–	–
Wu et al, 2010 (Chinese) [38]	NP >3 months	125	–	α = .89	ICC = .95	r_p_ = .81 NPDS r_p_ = -.76 to -.33 SF-36 subscales r_p_ = .75 VAS-P	–	–
Odole et al, 2011 (Nigerian) [35]	Mechanical NP	32	–	–	r = .97	–	–	–
Pickering et al, 2011 (English) [55]	Mechanical NP	88	1 factor: NP and dysfunction related to activities	–	–	–	–	–
Shakil et al, 2011 (Hindi) [39]	NP	148	–	α = .99	ICC = .98	–	–	–
Uthaikhup et al, 2011 (Thai) [40]	NP	181	1 factor	α = .85	ICC = .85	r_s_ = .64 VAS-P	MDC = 16.1 points SEM = 5.8 points	–
Monticone et al, 2012 (Italian) [21]	Chronic NSNP >12 weeks	101	2 factors: activity of daily living (F#1), pain and concentration (F#2)	α = .84 F#1: α = .82 F#2: α = .72	ICC = .85 (95 % CI.78–.89) F#1: ICC = .81 (95 % CI .73–.87) F#2: ICC = .83 (95 % CI .76–.88)	r_s_ = .69 NPDS r_s_ = .55 HADS-D r_s_ = .44 NRS r_s_ = .42 HADS-A	MDC = 3 points F#1: MDC = 1 F#2: MDC = 1	–
Kesiktas et al, 2012 (Turkish) [41]	NP	30/185	1 factor	α = .88	ICC = .87–1.0	r_s_ = .76 VAS-D r_s_ = .62 HADS-D r_s_ = .60 VAS-P r_s_ = .58 HADS-A	–	–
Jorritsma et al, 2012a (Dutch) [62]	Chronic NP	125	–	α = .83	–	r_p_ = .77 NPDS r_p_ = -.63–.34 SF-36 subscales r_p_ = .52 VAS-D r_p_ = .43 VAS-P	–	–
Jorritsma et al, 2012b (Dutch) [64]	Chronic NP	125	–	–	–	–	MDC: 8.4 points SEM: 3.0 points	MCID: 3.5 points (Spec .66; Sens .74) AUC = .75 (95 % CI .64–.87)
Nakamaru et al, 2012 (Japanese) [43]	NP (acute, subacute and chronic)	110	2 factors: activities, symptoms	α = .88	ICC = .91 (95 % CI.82–.85)	r_p_ = -.51–.25 SF-36 subscales	MDC = 6.8 points SEM = 2.9 points	–
Luksanapruksa et al, 2012 (Thai) [42]	Outpatients with NP	46	1 factor (activities)	α = .92	ICC = .99	r_P_ = .89 VAS-P r_P_ = -.43 SF-36	–	–
Ailliet et al, 2013 (Dutch) [44]	NP	338	RA: unidimensionality is achieved by removing item #6 concentration	–	–	r_p_ = .75 DASH	–	–
Guzy et al, 2013 (Polish) [45]	NSNP >7 weeks	95	2 factors	α = .82 F#1: α = .77 F#2: α = .73	ICC = .99 (95 % CI.98–.99) LoA = ±2.48	r_p_ = .55 VAS-P	MDC = 5.96 points SEM = 2.15 points SRM = 1.61	MCID = 6.5 points (Spec .81; Sens.90) AUC = .91 (95 % CI .84–.97)
Johansen et al, 2013 (Norwegian) [46]	NP	249	RA: unidimensionality is achieved by removing items #1 pain, #5 headache, and #9 sleep	–	–	r_p_ = .59 HSCL r_p_ = .58 NRS NDI-7: r_p_ = .56 NRS r_p_ = .43 TSK NDI-7: r_p_ = .54 HSCL	–	–
Shaheen et al, 2013 (Arabic) [47]	Neck complaints	65	2 factors: activity of daily living (F#1); pain and concentration (F#2)	α = .89 F#1: α = .86 F#2: α = .77	ICC_2,1_ = .96 (95 % CI .93–.97) F#1: ICC_2,1_ = .86 (95 % CI .52–.79) F#2: ICC_2,1_ = .77 (95 % CI .66–.85) r_p_ = .92	–	–	–
Walton & MacDermid, 2013 (English) [58]	Mechanical NP	316	RA: unidimensionality is achieved by removing items #1 pain, #3 lifting, #4 reading, #5 headache, and #9 sleep	–	ICC_2,1_ = .92 (95 % CI 85–.96) NDI-5: ICC_2,1_ = .94 (95 % CI.83–.86)	r_p_ = .71 NRS r_p_ = .64 PCS r_p_ = .53 TSK NDI-5: r_p_ = .71 PCS NDI-5: r_p_ = .67 NPRS NDI-5: r_p_ = .54 TSK	MDC_90_ = 4.5 points SEM = 1.95 points ES = .71 NDI-5: MDC_90_ = 2.7 points SEM = 1.15 points ES = .85	AUC = .76 (95 % CI .63–.89) NDI-5: AUC = .72 (95 % CI .59–.86)
Cramer et al, 2014 (German) [48]	Chronic NSNP >12 weeks	558	1 factor	α = .81	ICC = .81 (95 % CI.78–.83)	r_s_ = -.45–-0.30 SF-36 subscales	–	–
Johansen et al, 2014 (Norwegian) [89]	NP	255	–	α = .83–.91	ICC = .84 (95 % CI.72–.91)	–	MDC = 6.15 points SEM = 4.44 points	MCID = 8.3 points AUC = .70 (95 % CI .58–.82)
Swanenburg et al, 2014 (German) [52]	Outpatients with NP	49	2 factors	α = .96	ICC_(2,1)_ = .92 (95 % CI.84–.96)	r_s_ = .55 VAS-P	MDC = 6.16 points SEM = 2.22 points	–
Hung et al, 2015 (English) [56]	Neck complaints	865	RA: unidimensionality is achieved by removing items #2 personal care, and #5 headache	–	–	–	–	–
Ailliet et al, 2015 (Dutch) [90]	NP	337	–	–	ICC = .88 LoA = -5.02– + 5.60	–	MDC = 5.40 points SEM = 1.95 points	MCID = 4.50 points AUC = .85
Cruz, 2015 (Portuguese) [49]	Chronic NP (for at least 3 months)	113	1 factor	α = .95	ICC = .91 (95 % CI.87–.94) LoA = 1.59 ± 3.70	r_s_ = .49 NRS	–	–
Joseph et al, 2015 (Marathi) [50]	NP > 3 months	81	–	α = .97	ICC = .95	r = .95 VAS-P	–	–
Monticone et al, 2015 (Italian) [24]	Outpatients with chronic NP	200	–	–	–	–	ES = .66 SRM = 1.09 GRI = .70	MCID = 3.5 points AUC = .96 (spec .81; sens .98)
Bakhtadze et al, 2015 (Russian) [51]	Acute (≤12 weeks) or chronic (≥12 week) NP	80/109	2 factors	α = .83 F#1: α = .82 F#2: α = .66	ICC = .91 (95 % CI.86–.94)	r_s_ = .62 GRS-P	MDC = 5.4 points	
Pereira et al, 2015 (Portuguese) [91]	Chronic NP	108	–	–	–	–	MDC_95_ = 12 points SEM = 4.27 points	MCID = 5.5 points AUC = .59 (spec .57; sens .61)

α Cronbach’s alpha, *AUC* area under the curve, *CI* confidence interval, *DASH* disabilities of the arm, shoulder, and hand questionnaire, *DEPS* depression scale, *DRI* disability rating index, *ES* effect size, *Est* extension, *Flex* flexion, *GRI* Guyatt’s responsiveness Index, *GRS-P* 11-point numerical graphic rating scale for pain, *HADS-A* hospital anxiety and depression scale of anxiety, *HADS-D* hospital anxiety and depression scale of depression, *HSCL* Hopkins symptom checklist–25, *ICC* intraclass correlation coefficient, *k* Cohen's kappa, *LoA* limits of agreement, *MCID* minimal clinically important difference, *MDC* minimal detectable change, *MDC*_*90*_ minimal detectable change at the 90 % confidence level, *MDC*_*95*_ minimal detectable change at the 95 % confidence level, *NBQ* neck Bournemouth questionnaire, *NDI* neck disability index, *NP* neck pain, *NPDS* neck pain and disability scale, *NPQ* northwick park questionnaire, *NRS* numeric rating scale, *PCS* pain catastrophizing scale, *PET* problem elicitation technique, *r* correlation coefficient, *r*_*p*_ Pearson correlation coefficient, *r*_*s*_ Spearman correlation coefficient, *RA* Rasch analysis, *ROM* range of motion, *Rot* rotation, *SEM* standard error of measurement, *Sens* sensitivity, *SF-36 PF subscale* the medical outcomes study 36-item short-form health survey physical functioning subscale, *Spec* specificity, *SRM* standardized response mean, *TSK* tampa scale for kinesiophobia, *VAS-A* visual analogue scale for activity, *VAS-D* visual analogue scale for disability, *VAS-P* visual analogue scale for pain

**Table 3:** Psychometric properties of the neck pain and disability scale

Authors, year (language)	Population	Sample size	Dimensionality	Internal consistency	Reliability	Validity	Responsiveness	
							Distribution-based methods	Anchor-based methods
Wheeler et al, 1999 (English) [65]	NP	100	4 factors: neck problems; pain intensity; effect of neck pain on motion and cognition; neck pain interference with life activities	α = .93	–	r_p_ = .78 ODI r_p_ = .80 PDI r_p_ = .52 BDI	–	–
Goolkasian et al, 2002 (English) [73]	NP	83	–	–	r_p_ = .93	r = .59 Patient GAS r = .59 Physician GAS r = .74 PDI r = .72 NDI	–	–
Wlodyka-Demaille et al, 2002 (French) [28]	NP >15 days	101	3 factors: function and disability; neck pain intensity during movement; static neck pain intensity	–	ICC = .91	r_s_ = .79 NDI r_s_ = .73 NPQ r_s_ = .63 VAS-D r_s_ = .51 VAS-P r_s_ = .40 HADS-A r_s_ = .49 HADS-D r_s_ = -.45 ROM (Flex-Ext)	–	–
Bicer et al, 2004 (Turkish) [69]	NP >6 months	61	–	α = .86 Item to total correlation from .08 to .69	–	r = .51 PDI r = .45 VAS-P	–	–
Wlodyka-Demaille et al, 2004 (French) [75]	NP >15 days	71	–	–	–	–	ES = .46 SRM = .38	–
Lee et al, 2006 (Korean) [29]	NP	180	–	α = .96	ICC = .90(95 % CI .83–.95)	–	ES = 1.07 SRM = 1.34	AUC = .79 (95 % CI 72–.86)
Kose et al, 2007 (Turkish) [31]	NP >6 weeks	102	–	α = .94	ICC = .81	r = .40 Morning stiffness r = .73 VAS-P r = .69 VAS-D r = .46 VAS-Physician’s assessment r = .49 Pain with motion	SRM = .89–.92	–
Mousavi et al, 2007 (Iranian) [32]	NP	185	4 factors: neck dysfunction and disability (F#1), neck pain intensity (F#2), neck pain during movement (F#3), static neck pain problems (F#4)	F#1: α = .94 F#2: α = .92 F#3: α = .84 F#4: α = .75	F#1: ICC = .95 F#2: ICC = .97 F#3: ICC = .92 F#4: ICC = .90	F#1: r_p_ = -.69 to -.40 SF-36 subscales F#2: r_p_ = -.57 to -.24 SF-36 subscales F#3: r_p_ = -.54 to -.17 SF-36 subscales F#4: r_p_ = -.63 to -.18 SF-36 subscales F#1: r_p_ = .63 VAS-P F#2: r_p_ = .77 VAS-P F#3: r_p_ = .79 VAS-P F#4: r_p_ = .46 VAS-P Total NPDS: r_p_ = .86 NDI	–	–
Monticone et al, 2008 (Italian) [20]	NP subacute (pain >4 weeks) and chronic (pain >12 weeks)	157	3 factors: neck dysfunction related to general activities (F#1), neck pain and cognitive-behavioral aspects (F#2), neck dysfunction related to activities of the cervical spine (F#3)	Total NPDS: α = .94 F#1: α = .92 F#2: α = .86 F#3: α = .89	Total NPDS: r_s_ = .91 F#1: r_s_ = .89 F#2: r_s_ = .93 F#3: r_s_ = .92	r_P_ = -.47 SF-36 r_P_ = -.45 to -.17 SF-36 subscales	–	–
Scherer et al, 2008 (German) [67]	NP	448	3 factors	α = .94 Item-to-total correlation from .46 to .82	–	r_p_ = .44 HADS-D r_p_ = .41 HADS-A	–	–
Chan et al, 2009 (English) [74]	NP	20	–	–	–	r_p_ = .71 PET r_p_ = .86 NDI	–	–
Jorritsma et al, 2010 (Dutch) [70]	NP >3 months	33	–	–	ICC = .76 (95 % CI .57–.87) LoA: ±20.9	–	–	–
Wu et al, 2010 (Chinese) [38]	NP >3 months	125	3 factors: neck disfunction and disability (F#1), neck pain intensity during movement (F#2), static neck pain intensity (F#3)	F#1: α = .91 F#2: α = .88 F#3: α = .82	F#1: ICC = .94 F#2: ICC = .92 F#3: ICC = .86	Total NPDS: r_p_ = .81 NDI F#1: r_p_ = -.61 to -.20 SF-36 subscales F#2: r_p_ = -.58 to -.19 SF-36 subscales F#3: r_p_ = -.61 to -.22 SF-36 subscales F#1: r_p_ = .72 VAS-P F#2: r_p_ = .62 VAS-P F#3: r_p_ = .76 VAS-P	–	–
Blozik et al, 2011 (German) [72]	NP	411	–	–	–	–	MDC = 3 points SEM = .9 points SRM = 1.1	–
Chen et al, 2011 (Chinese) [68]	NP >3 months	106	4 factors: pain (F#1), disability (F#2), Neck specific function (F#3), emotional and cognitive influences (F#4)	Total NPDS: α = .97 F#1: α = .93 F#2: α = .95 F#3: α = .95 F#4: α = .91 Item-to-total correlation: from .68 to .88	Total NPDS: r = .81 F#1: r = .89 F#2: r = .97 F#3: r = .91 F#4: r = .94	Total NPDS: r_p_ = -.72 SF-36 Total NPDS: r_p_ = -.71 to -.12 SF-36 subscales F#1: r_p_ = -.74 SF-36 F#1: r_p_ = -.71 to -.05 SF-36 subscales F#2: r_p_ = -.67 SF-36 F#2: r_p_ = -.65 to -.14 SF-36 subscales F#3: r_p_ = -.49 SF-36 F#3: r_p_ = -.65 to -.04 SF-36 subscales F#4: r_p_ = -.63 SF-36 F#4: r_p_ = -.63 to -.12 SF-36 subscales	–	–
Ono et al, 2011 (Japanese) [71]	NP	167	2 factors: neck-pain-related disability (F#1) and neck-related pain (F#2)	Total NPDS α = .96 F#1: α = .94 F#2: α = .93	Total NDPS: ICC = .77 F#1: ICC = .75 F#2: ICC = .77	Total NPDS: r_p_ = -.54 to -.24 SF-36 subscales F#1: r_p_ = -.50 to -.24 SF-36 subscales F#2: r_p_ = -.51 to -.22 SF-36 subscales Total NPDS: r_p_ = .71 VAS-P F#1: r_p_ = .56 VAS-P F#2: r_p_ = .77 VAS-P Total NPDS: r_p_ = .59 VAS-D F#1: r_p_ = .48 VAS-D F#2: r_p_ = .63 VAS-D	–	–
Pickering et al,, 2011 (English) [55]	NP	88	3 factors: dysfunction related to general activities and the impact of participation restriction on psychosocial function; neck pain and interference with neck-specific function; cognitive and emotional functioning	–	–	–	–	–
Uthaikhup et al, 2011 (Thai) [40]	NP	172	3 factors: disability (F#1), pain (F#2) neck specific function (F#3)	Total NDPS: α = .96 F#1: α = .94 F#2: α = .90 F#3: α = .92	Total NDPS: ICC = .88 F#1: ICC = .81 F#2: ICC = .91 F#3: ICC = .74	Total NPDS: r_s_ = .76 VAS-P F#1: r_s_ = .74 VAS-P F#2: r_s_ = .65 VAS-P F#3: r_s_ = .61 VAS-P	MDC = 21.8 points SEM = 7.9	–
Jorritsma et al, 2012a (Dutch) [62]	NP	125	–	α = .93 Item-total correlations from .45 to .73	–	r_p_ = -.70 to -.36 SF-36 subscales r_p_ = .54 VAS-P r_p_ = .57 VAS-D r_p_ = .77 NDI	–	–
Jorritsma et al, 2012b (Dutch) [64]	NP	125	–	–	–	–	MDC = 31.7 points SEM = 11.4	MCID = 11.5 points (Sens .74; Spec .70) AUC = .75 (95 % CI .62–.87)
Monticone et al, 2015 (Italian) [24]	NP	200	–	–	–	–	ES = .73 SRM = 1.26 GRI = .73	MCID = 10 points AUC = .91 (Sens .93; Spec .83)

*α* Cronbach’s alpha, *AUC* area under the curve, *BDI* Beck depression inventory, *CI* confidence interval, *Flex* flexion, *ES* effect size, *Ext* extension, *GAS* global assessment score, *GRI* Guyatt’s responsiveness index, *HADS-A* hospital anxiety and depression scale of anxiety, *HADS-D* hospital anxiety and depression scale of depression, *ICC* intraclass correlation coefficient, *LoA* limits of agreement, *MCID* minimal clinically important difference, *MDC* minimal detectable change, *NDI* neck disability index, *NP* neck pain, *NPDS* neck pain and disability scale, *NPQ* northwick park questionnaire, *ODI* oswestry disability index, *PDI* pain disability index, *PET* problem elicitation technique, *r* correlation coefficient, *r*_*p*_ Pearson’s correlation coefficient, *r*_*s*_ Spearman’s correlation coefficient, *ROM* range of motion, *SEM* standard error of measurement, *Sens* sensibility, *SF-36* the medical outcomes study 36-item short-form health survey, *Spec* specificity, *SRM* standardized response mean, *VAS-D* visual analogue scale for disability, *VAS-H* visual analogue scale for global health, *VAS-P* visual analogue scale for pain

**Table 4:** Psychometric properties of the neck bournemouth questionnaire

Authors, year (language)	Population	Sample size	Dimensionality	Internal consistency	Reliability	Validity	Responsiveness	
							Distribution-based methods	Anchor-based methods
Bolton & Humphreys, 2002 (English) [76]	NP	102	–	α = .87–.92	ICC = .65	r = .50–.71 NDI r = .44–.63 CNFDS	ES = 1.67 SRM = 1.43	–
Bolton, 2004 (English) [80]	NP	71	–	–	–	–	ES = 1.67 SRM = 1.01	RCI (>1.96) = 13 points
Gay et al, 2007 (English) [59]	Chronic NP > 3 months	23	–	α = .85–.89	–	r = .77–.80 NDI r = .37–.62 VAS-P	ES = 1.28 SRM = 1.17	–
Martel et al, 2009 (French) [77]	Chronic NP	68	–	–	r = .97 (95 % CI95–.98) ICC = .97 (95 % CI.95–.98)	r = .61–.67 NDI	ES = .56 SRM = .61	RCI (>1.96) = 4.4 points
Soklic et al, 2012 (German) [78]	NP	102	–	α = .79–.82	ICC = .99 (95 % CI.98–.99)	r = .68–.76 NDI r = .69–.80 NPDS	SRM = .73–1.20	–
Geri et al, 2014 (Italian) [23]	Chronic NP >3 months	96	2 factors: pain & functioning (F#1); anxiety & depression (F#2)	Total score: α = .89 (95 % CI.84–.92) F#1: α = .88 (95 % CI.83–.92) F#2:α = .90 (95 % CI.86–.94)	–	r = .67–.70 NPDS r = .63–.73 NRS	–	MCID = 5.5 points AUC = .72 (Sens. 75 %; Spec. 60 %)
Geri et al, 2015 (Italian) [79]	Chronic NP	161	2 factors: pain & functioning (F#1); anxiety & depression (F#2)	PSI (F#1) = .80 PSI (F#2) = .77	–	–	–	–

*α* Cronbach’s alpha, *AUC* area under the curve, *CI* confidence interval, *CNFDS* copenhagen neck functional disability scale, *ES* effect size, *ICC* intraclass correlation coefficient, *MCID* minimal clinically important difference, *NDI* neck disability index, *NP* neck pain, *NPDS* neck pain and disability scale, *NRS* numerical rating scale, *PSI* person separation index, *r* correlation coefficient, *RCI* reliable change index, *Sens* sensibility, *Spec* specificity, *SRM* standardized response mean, *VAS-P* visual analogue scale for pain

**Table 5:** Psychometric properties of the core outcome measure index

Authors, year (language)	Population	Sample size	Dimensionality	Internal consistency	Reliability	Validity	Responsiveness	
							Distribution-based methods	Anchor-based methods
White et al., 2004 (English) [81]	Chronic mechanical NP	133	–	–	Single items: ICC = .64–.99	Pain: r = .73 VAS-P Other items: r = .60 NDI	–	–
Kovacs et al, 2008 (Spanish) [82]	Acute, subacute and chronic NP	167	–	Pain: α = .73 Disability: α = .84	ICC = .85 (95 % CI .75–.91)	r_p_ = .61 VAS-P r_p_ = .46 VAS-referred P r_p_ = .57 CSQ r_p_ = .69 NDI r_p_ = .71 NPQ r_p_ = -.60 SF-12 PF	Pain: ES = .79 Disability ES = .92	–
Miekisiak et al, 2014 (Polish) [83]	NP >4 weeks	123	1 factor	–	ICC = .88 (95 % CI .82–.92)	r_s_ = .62 NDI	MDC = 2/10 points SEM = .7/10 points	–
Monticone et al, 2014 (Italian) [22]	Chronic NP >3 months	103	–	–	ICC = .87 (95 % CI .81–.91)	Pain: r_p_ = .45 NRS Pain: r_p_ = .48 NPDS Function: r_p_ = .49–.55 NPDS QoL: r_p_ = -.44 EQ-5D Disability: r_p_ = .45–.48 NPDS	MDC = 1.8/10 points SEM = .65/10 points SRM = 1.23	AUC: .73 (.62–.85) (Sens = .55; Spec = .88)

*α* Cronbach’s alpha, *AUC* area under the curve, *CI* confidence interval, *CSQ* coping strategies questionnaire, *EQ-5d* Euroqol 5-dimensions, *ES* effect size, *ICC* intraclass correlation coefficient, *MDC* minimum detectable change, *NDI* neck disability index, *NP* neck pain, *NPDS* neck pain and disability scale, *NPQ* northwick park questionnaire, *NRS* numeric rating scale, *QoL* quality of life, *r* correlation coefficient, *r*_*p*_ Pearson’s correlation coefficient, *r*_*s*_ Sperman’s correlation coefficient, *SEM* standard error of measurement, *Sens* sensitivity, *SF-12 PF* 12-item short-form health survey physical functioning subscale, *Spec* specificity, *SRM* standardized response mean, *VAS-P* visual analogue scale for pain

**Table 6:** Psychometric properties of the NeckPix®

Authors, year (language)	Population	Sample size	Dimensionality	Internal consistency	Reliability	Validity	Responsiveness	
							Distribution-based methods	Anchor-based methods
Monticone et al, 2014 (Italian) [84]	Chronic NP (>12 weeks)	118	1 factor	α = .95	ICC = .98 (95 % CI .97–.98)	r_p_ = .76 TSK r_p_ = .58 PCS r_p_ = .52 NDI r_p_ = .45 NRS	–	–

*α* Cronbach’s alpha, *CI* confidence interval, *ICC* intraclass correlation coefficient, *NP* neck pain, *NDI* neck disability index, *NRS* numeric rating scale, *PCS* pain catastrophizing scale, *r*_*p*_ Pearson’s correlation coefficient, *TSK* tampa scale for kinesiophobia

#### Neck disability index

The NDI [25] was adapted from an existing questionnaire for low back pain (the Oswestry Disability Index) to assess neck pain and disability. It contains ten items exploring pain intensity, personal care, lifting, reading, headaches, concentration, work, driving, sleeping and recreation. Each item is scored from 0 (no disability) to 5 (worst disability). The total score is calculated by adding the scores of each item and ranges from 0 to 50, although it is also frequently normalized to 100 or reported as a percentage. The NDI has been translated into many languages [26–52], including Italian [21] (Table 2). The time needed to administer the questionnaire is about 5 to 10 min [21, 28, 36, 41, 51].

Different opinions exist on what the NDI aims to measure and how scores should be interpreted. Although the NDI was mostly considered as a one-factor measure of functional status [33, 34, 37, 40–42, 48, 49, 53–55], other studies [28, 43, 45, 47, 52] -including two of excellent methodological quality [21, 51] - suggested the likely presence of sub-dimensions and considered the scale as a measure of pain and disability. According to RA, to achieve unidimensionality some items would need to be removed, but there is no agreement about which (and how many) to remove [44, 46, 56–58]. For example, Johansen et al. [46] proposed a 7-item NDI with a single underlying dimension of disability. They claimed that after removing body function items (#1 pain, #5 headache, and #9 sleep problems), the remaining items - representing the International Classification of Functioning Disability and Health (ICF) component of Activities and Participation - fitted the Rasch model. Suggestions for item reduction ranged from 1 [44] to 5 items [58].

The raw score to measure correlation was poor, indicating that summing of the raw scores is not acceptable and meaningful [56]. The NDI raw score is not linear, and it does not carry with it a clear interpretation of what a score means. Internal consistency was found to be high, ranging from 0.72 [59] to 0.99 [39]. The questionnaire proved to be reliable in most (with ICC values ranging from 0.81 to 0.99) [27, 45, 48] but not all studies [60, 61], that reported very low reliability values. All of these studies were of poor to fair quality and no firm conclusions can be drawn.

The NDI total score showed moderate to strong correlations with the Visual Analogue Scale for pain (VAS) [28, 31, 32, 34, 38, 42, 50, 53], Numeric Rating Scale (NRS) [46, 58], Short Form-36 (SF-36) subscales [27], and other neck disability questionnaires such as NBQ [59] and NPDS [21, 32, 38, 62]. A ceiling and a floor-ceiling effect was also reported [30, 53, 56].

Responsiveness was highly affected by the measurement error, as shown also by the very low reliability values reported [60, 61]. Anchor-based methods gave a MCID ranging from 3.5 [63, 64] (including one study from Italy of excellent quality [24]) to 9.5 [60] points on a 50-point scale, but the MDC_95_ showed a very large variability ranging from 1.66 [30] to 23.3 points [60] in studies of fair quality. Accordingly, the amount of change perceived as important by patients is less than 20 % of the maximal total score, but the error of the scale can theoretically reach nearly 50 % of the score.

#### Neck pain and disability scale

The NPDS was developed [65] to measure neck pain and disability using the Million Visual Analogue Scale [66] as a template. It consists of 20 items measuring the intensity of pain, its interference with vocational, recreational, social and functional aspects of living, and the presence and extent of associated emotional factors. Each item is rated from 0 to 5 on a 10 cm VAS divided into 5 equal intervals by vertical bars. Midpoints for each interval are marked with two dots. The total NPDS score is the sum of the scores for all 20 items, ranging from 0 (no disability) to 100 (greatest disability). The maximum acceptable number of missing answers is 4 [67, 68]. The NPDS has been validated in several languages [28, 29, 31, 32, 38, 40, 67–71], including Italian [20] (Table 3).

Factor analysis revealed either two [71], three [20, 28, 38, 40, 55, 67], or four factors [38, 65, 68], but the items constituting each factor were not consistent across studies of comparable quality. The average time to complete the questionnaire was reported to be generally lower than 8 min [20, 28, 65].

Internal consistency was high, with Cronbach’s alpha for the total score ranging from 0.86 [69] to 0.97 [68]. The ICC values were above 0.75, but only in a few studies of lower quality [20, 28, 32, 38, 73] did they exceed the minimum required value of 0.90.

The NPDS showed a strong correlation with concurrent scales such as the NDI [28, 32, 62, 73, 74] and the Northwick Park Questionnaire (NPQ) [28], moderate to strong correlations with VAS pain [28, 31, 38, 40, 69, 71], and a weak to moderate correlation with SF-36 [20, 32, 38, 71]. The NPDS demonstrated good face validity, being able to discriminate (*p* <.01) patients with neck pain from healthy subjects or subjects with low back and leg pain [65]. Content validity was confirmed by the high rate of answers to all items, while the most common missing items concerned driving, reading, and medication [32, 40, 70, 74]. There were no floor or ceiling effects found [28, 29, 32, 40, 63, 72, 75].

The ES and SRM values reported varied widely across studies. Because these indices are based on standard deviations, the differences observed may be due to the sample size or patient selection of the studies. Similarly, the different methods adopted to calculate the MDC across studies led to very different results in the studies of poor quality, ranging from 3 [72] to 31.7 points [64]. The MCID was close to 10 points both for the Italian version in a study of excellent quality (AUC 0.91; sensitivity 0.93; specificity 0.83) [24] and for the Dutch version in a low quality study (11.5 points; AUC 0.75; sensibility 0.74; specificity 0.70) [64].

#### Neck Bournemouth questionnaire

The NBQ is a self-report questionnaire developed to measure neck pain according to the biopsychosocial model [76]. It consists of 7 items rated on a NRS from 0 to 10 (where 0 means ‘much better’, 5 ‘no change’, and 10 ‘much worse’) for a total score range 0–70, with higher scores reflecting more severity. The NBQ has been translated into several languages, including French [77], German [78], and Italian [23] (Table 4).

Factor analysis was conducted on the Italian version in a good quality study, and revealed a model composed of two different subscales dealing with pain & functioning (factor 1, items #1, #2, #3, #6, and #7, explaining 56.6 % of the variance), and anxiety & depression (factor 2, item #4 and #5, explaining 12.6 % of the variance) [23]. Cronbach’s alpha for the total score ranged from 0.79 [78] to 0.92 [76], indicating a high interrelatedness of the items with a possible tendency to redundancy. The internal consistency of the two subscales revealed a similar pattern [23]. Confirmatory factor analysis indicated item #7 as unnecessary in factor 1, while for factor 2 the high redundancy could be attributable to the overlapping of feelings like anxiety and depression [23]. A recent Rasch Italian study [79] confirmed the presence of two factors. After removal of item #7, the first factor (pain & functioning) fitted the Rasch model, while the second factor (anxiety & depression) fitted the model without modification. The time needed to complete the questionnaire is less than 5 min [23, 76]. Test-retest reliability ranged from moderate [76] to excellent [77, 78].

The NBQ showed a moderate to strong correlation with most existing questionnaires, such as NDI [59, 76–78], NPDS [23, 78], and the Copenhagen Neck Functional Disability Scale [76], but a weak to moderate correlation with VAS pain [59]. A large portion of patients judged the NBQ as relevant to their health problem (78.7 %) or as relevant for other people with neck pain (87.9 %) [79], confirming the face validity of the questionnaire. A floor effect (19.4 % of patients attained the lowest score) was observed in the anxiety and depression factor’s score after treatment [79].

The NBQ was considered a sensitive outcome measure able to depict moderate-to-large change in groups of patients with NSNP. The MCID was estimated using both ROC and Reliable Change Index methods. Two studies of fair to good quality reported similar findings, ranging from 4.4 [77] to 5.5 points [23], but higher raw change scores of 13 points or more (and percentage change scores of 36 % or more) were also reported in a study of poor quality as giving the best balance between sensitivity and specificity in detecting clinically improved patients [80]. The MDC of the questionnaire has never been calculated.

#### Core outcome measures index for neck pain

This questionnaire was adapted with some minor changes from the existing low back pain version. It contains seven items pertaining to five domains: severity of pain, function, symptom-specific well-being, quality of life, and disability (social and work). Items refer to how the subject felt in the last week, except for those regarding disability which refer to the last month. Pain items use a 0–10 cm VAS and the higher of the two scores is used to represent pain. The other items use a 5-point Likert-type scale. The COMI score is calculated by averaging the values for each domain (with higher scores indicating a worse status) into a 0-5 score [81, 82] or - more recently - after re-scoring them on a 0–10 scale [22, 83]. The COMI has been translated into Spanish [82], Polish [83], and Italian [22] (Table 5). The time required to complete the questionnaire is less than 3 min and the acceptability was found to be good, as shown by the absence of problems in comprehension or of missing or multiple answers [22].

Factorial analysis was performed only on the Polish version in a study of excellent methodological quality [83], and a single factor explaining 61.6 % of the variation in score was identified. Internal consistency was measured only for the pain and disability subscales with acceptable values in a poor quality study [82], and the test-retest reliability of the total score was almost high [23, 82]. The COMI total score was found to be consistent with the external criterion for disability (values increased as patients’ self-perception of disability increased), but not with that for pain [82]. The COMI showed a lower correlation than other questionnaires (e.g. NDI and NPQ) with measures of pain or disability. The Italian [23] and Polish [83] versions showed also some floor and ceiling effects.

The COMI was found to be poorly sensitive to worsening of both pain and disability; it reflected improvement in pain for patients who denied any change, and it magnified the amount of improvement for pain and, especially, for disability [82]. MDC values were about 2/10 points for both the Italian [23] and Polish versions [83] in good quality studies. The ROC analysis was carried out on the COMI change scores in a study of poor methodological quality, revealing a significant ability to discriminate poor from good patients, with the cut-off set at two points [23].

#### NeckPix©

This measure [84] was recently developed in Italian to assess activity-related kinesiophobia in outpatients with chronic NSNP (Table 6). It consists of ten images that represent everyday activities involving the neck. The patient rates from 0 to 10 (0 = no fear, 10 = greatest fear) the fear of feeling pain in the neck when doing the activity represented in each image. The total score ranges from 0 to 100. The scale requires a mean time of 2 min to complete.

An excellent methodological quality exploratory factor analysis revealed a one-factor structure [84]. The internal consistency and reliability were excellent, and good correlations were found with the Tampa Scale of Kinesiophobia and the Pain Catastrophizing Scale. No floor or ceiling effects were observed.

## Discussion

Four instruments measuring function and disability, and one measuring activity-related fear of movement, are now available for assessing Italian people with non-specific neck pain. In 2011, a systematic review [5] of translated versions of neck-specific questionnaires was able to identify only one instrument. Overall, the available information on measurement properties of the Italian versions of PROMs for NSNP are good, despite the poor methodological quality of most translations.

### Psychometric properties

Among the instruments considered in this review, the NDI is the one that has been most widely studied. It is the only instrument having all the measurement properties validated and with positive findings [4, 5]. However, important issues regarding dimensionality and responsiveness emerged. Factor analysis raised uncertainty about the presence of a single construct, which was definitively rejected by RA [44, 46, 56–58]. Unidimensionality could be achieved by removing from 1 [44] to 5 [58] of the 10 original items. While item #5 (headache) was a common misfitting item (headache may not be a common symptom experienced by all neck pain patients, and therefore not sensitive to change) [57], there was no consistency between studies on which items exactly should be removed. The NDI showed also a large floor effect [56]. As a result, the NDI may be inadequate to assess patients with moderate to high functioning, and it may not be sensitive to changes in patients’ functioning over time. Problems with responsiveness were also related to the large variability of measurement error [30, 60], and a poor raw score to measure correlation was found [56]. Before adopting the NDI as the instrument of first choice and determining a range for MCID, the dimensionality, reliability and measurement error of this questionnaire needs to be carefully assessed.

The NPDS was the first instrument translated into Italian, and its measurement properties have been extensively examined. However, agreement on its dimensionality is still lacking. The developers originally described a 4-factor structure, but the Italian validation study extracted only three factors. The high variability among studies precludes any confident judgement about the factorial structure and content of the scale. This raises the need for RA to test its dimensionality and metrics before it can be recommended to interpret clinical changes in individual patients. Future studies should also carefully estimate the measurement error, to verify that it does not exceed the MCID.

The NBQ demonstrated acceptable psychometric properties when tested with CTT methods. The results of both factor analysis and RA revealed a robust 2-factor structure [23, 79], and a refined version with removal of item #7 was proposed [79]. This implies that two independent subscales should be used in place of a total composite score. Subscale 1 was intended to measure neck-related disability (similar to that of the NDI) and was better suited to assess the health status of patients with chronic NSNP in research settings [79]. Subscale 2, dealing with anxiety & depression, should be used with caution given the presence of only two items. To avoid biased conclusions about treatment effectiveness, it was recommended to use the Rasch-conversion tables provided for each subscale of the Italian version [79]. The responsiveness should be also re-assessed taking into consideration the deletion of item #7 from subscale 1. After that, the NBQ could be considered a valid instrument to measure quality of life in people suffering from NSNP.

The COMI has been less extensively studied than the instruments above, and some problems regarding the sensitivity to change have emerged. The exploratory factor analysis showed a mono-factorial structure, but the paucity of information about the dimensionality of this scale warrants further investigation with RA. Inconsistencies between studies also emerged in this review, in particular concerning the methods used to calculate the total score, the classification of items, and the scoring categories of some items. This could lead to misunderstandings when comparing results across studies.

The NeckPix© - recently developed in Italy - showed a robust factorial structure and good reliability and validity. However, no information about its responsiveness was provided by the developers. It constitutes an innovative and promising measure of activity-related kinesiophobia, but before it can be recommended as an outcome measure for clinical and research purposes, this instrument needs to undergo further research to confirm its measurement properties and clarify how to interpret the results.

### Clinical utility

Among the PROMs with comparable validity, reliability and responsiveness, the choice of which measurement tool to use should be made only after a careful evaluation of the clinical utility, and depends on what type of intervention is planned and what the anticipated response is. The clinical utility of a measure relates to its ease and efficiency of use, and to the relevance and meaningfulness of the information that it provides [85]. No substantial differences in core elements such as ease of use, time taken to administer, training and qualification of clinicians required, format (acceptability), and cost were observed between the instruments evaluated in this study. On the other hand, differences emerged as to their content (i.e. which domains the PROMs are intended to measure), and this may be of greater interest to clinicians who need to make a precise assessment of specific aspects that affect patients with NSNP. The content of NeckPix© is appropriate for evaluating activity-related fear of movement, while the other four instruments are aimed at measuring mainly function and disability, and could be classified using the ICF [86] framework. The ICF identifies two different relevant domains that should be addressed: 1) Functioning, Disability and Health, which includes: i) Body Functions, ii) Body Structures, iii) Activity and Participation; and 2) Contextual Factors, that include: i) Environmental Factors, and ii) Personal Factors [87]. As there is currently no core set of domains for neck pain assessment, the patient’s own experience has been used to classify their functional problems and these have been linked to the ICF. Problems with functioning belonging to the Activities and Participation component (such as computer work, driving, maintaining a body position, lifting and carrying objects) were the most frequently reported [88]. However, patients with neck problems reported also a higher proportion of body function impairments (such as sleep disturbance, functional problems with mobility of joint functions) than patients with musculoskeletal pain in other body regions [87]. That indicates a multidimensionality of their functional problems, and requires an in-depth assessment.

For the purposes of the present study, PROMs were linked to the ICF framework within the components described above. However, coding questionnaires is not always straightforward: items of each instrument could be linked across more than one category, or may not be classified at all. The NDI had four items (40 %) categorized as body functions, and six items classified as activity and participation (60 %); the NPDS contained 11 items (55 %) classified as body functions, eight in the activity and participation category (40 %), and 1 (5 %) pertaining to environmental factors; the NBQ had three items (43 %) classified as body functions and 3 (43 %) as activity and participation (one item could not be classified into the ICF categories); the COMI had two items (33 %) classified as body functions and 4 (67 %) as activity and participation. All four instruments showed a well-balanced distribution of items across the body functions and activity and participation components, although in different ratios and with a different ICF category coverage. For example, the NPDS is the only one that assesses contextual factors such as drug use.

NSNP is a complex, multidimensional experience and it is imperative that PROMs assess and reflect this accurately, in order to be useful in both the clinical and research settings. Multimodal interventions may be more effectively measured by a scale that can be demonstrated to measure a variety of factors that contribute to neck pain and related disability. However, the disadvantage of using multidimensional scales is that interpreting the meaning of the overall score and determining the attribution of changes becomes more difficult.

### Limitations

The search was restricted to studies published in English and Italian. However, as the aim of this review was to identify the PROMs validated in Italian, the likelihood of further relevant articles published in different languages was very low. It should also be noted that this study examined those PROMs aimed to evaluate patients with NSNP only, so data extracted from other samples (e.g. in patients with whiplash or after neck surgery) were excluded. The risk of bias of the studies included in this review was not assessed, as most information was considered from studies at low risk of bias.

## Conclusions

In the last 5 years, four instruments (NDI, NPDS, NBQ, and COMI) have been translated into Italian language with the aim to measure function and disability and one (NeckPix©) to measure activity-related fear of movement. The most widespread PROM is the NDI, but important issues about its dimensionality and responsiveness emerged, especially in patients with moderate to high functioning. The NPDS has also been extensively investigated, but the agreement on its dimensionality is still lacking. The NBQ has demonstrated good psychometric properties, especially in the Italian version. If they are confirmed by further studies, this scale could be considered as a comprehensive tool for measuring pain & functioning, and anxiety & depression in patients with NSNP.

## Abbreviations

AUC, area under curve; COMI, core outcome measures index; COSMIN, COnsensus-based Standards for the selection of health Measurement INstruments; CTT, classical test theory; ES, effect size; GRI, Guyatt’s responsiveness index; ICC, intraclass coefficient correlation; ICF, international classification of functioning disability and health; MCID, minimal clinically important difference; MDC, minimum detectable change; NBQ, neck Bournemouth Questionnaire; NDI, neck disability index; NPDS, neck pain and disability scale; NPDS, neck pain and disability scale; NPQ, northwick park questionnaire; NRS, numeric rating scale; NSNP, non-specific neck pain; PROM, patient-reported outcome measure; RA, Rasch analysis; ROC, receiver operating characteristic; SF-36, medical outcomes study 36-item short-form health survey; SRM, standardized response mean; VAS, visual analogue scale

## Appendix

### Search strategies for each investigated database

#### MEDLINE

(“Psychometrics”[Mesh] OR “Outcome Assessment (Health Care)”[Mesh] OR “Validation Studies as Topic”[Mesh] OR “Validation Studies”[Publication Type] OR “Questionnaires”[Mesh] OR “Evaluation Studies”[Publication Type] OR “Translations”[Mesh] OR “Translating”[Mesh] OR “Cross-Cultural Comparison”[Mesh] OR “Reproducibility of Results”[Mesh] OR valid* OR accuracy OR reliability OR agreement OR reproducibility OR “sensitivity to change” OR responsiveness OR “minimal detectable change” OR “minimal clinical* important change” OR “minimal clinical* important difference” OR “minimal important change” OR “floor effect” OR “ceiling effect” OR “factor analysis” OR translation OR rasch OR psychometrics OR version) AND (“Neck Pain”[Mesh] OR “neck pain” OR “cervical pain”).

#### EMBASE

(‘outcome assessment’/exp OR ‘outcome assessment’ OR ‘validation studies’/exp OR ‘validation studies’ OR ‘questionnaires’/exp OR questionnaires OR ‘evaluation studies’/exp OR ‘evaluation studies’ OR ‘translati*’ OR ‘cross-cultural validation’ OR valid* OR ‘accuracy’/exp OR accuracy OR ‘reliability’/exp OR reliability OR agreement OR ‘reproducibility’/exp OR reproducibility OR ‘sensitivity to change’ OR responsiveness OR ‘minimal detectable change’ OR ‘minimal clinical* important change’ OR ‘minimal clinical* important difference’ OR ‘minimal important change’ OR ‘floor effect’ OR ‘ceiling effect’ OR ‘factor analysis’/exp OR ‘factor analysis’ OR translation OR rasch OR ‘psychometrics’/exp OR psychometrics OR version) AND (‘neck pain’/exp OR ‘neck pain’ OR ‘cervical pain’).

#### CINHAL

(“Outcome Assessment” OR “Validation Studies” OR Questionnaires OR “Evaluation Studies” OR Translati* OR “Cross-Cultural Validation” OR valid* OR accuracy OR reliability OR agreement OR reproducibility OR “sensitivity to change” OR responsiveness OR “minimal detectable change” OR “minimal clinical* important change” OR “minimal clinical* important difference” OR “minimal important change” OR “floor effect” OR “ceiling effect” OR “factor analysis” OR translation OR rasch OR psychometrics OR version) AND (“Neck Pain” OR “cervical pain”).

#### Scopus, Web of Science, Cochrane Library

(Questionnaires OR validity OR accuracy OR reliability OR agreement OR reproducibility OR “sensitivity to change” OR responsiveness OR “factor analysis” OR translation OR rasch OR psychometrics OR version) AND (“Neck Pain” OR “cervical pain”).
